# Identification and profiling of microRNAs during yak’s testicular development

**DOI:** 10.1186/s12917-023-03602-7

**Published:** 2023-02-20

**Authors:** Yongfu La, Xiaoming Ma, Pengjia Bao, Min Chu, Xian Guo, Chunnian Liang, Ping Yan

**Affiliations:** 1 Animal Science Department, Lanzhou Institute of Husbandry and Pharmaceutical Sciences, Chinese Academy of Agricultural Sciences, Lanzhou, China; 2 Key Laboratory of Animal Genetics and Breeding On Tibetan Plateau, Ministry of Agriculture and Rural Affairs, Chinese Academy of Agricultural Sciences, Lanzhou, China; 3 Key Laboratory for Yak Genetics, Breeding, and Reproduction Engineering of Gansu Province, Chinese Academy of Agricultural Sciences, Lanzhou, China

**Keywords:** Testis, Yak, MicroRNA, Development, Spermatogenesis

## Abstract

**Background:**

Normal testicular development is highly crucial for male reproduction and is a precondition for spermatogenesis that is the production of spermatozoa in the testes. MiRNAs have been implicated in several testicular biological processes, including cell proliferation, spermatogenesis, hormone secretion, metabolism and reproductive regulation. In the present study, we used deep sequencing data to study the functions of miRNAs during testicular development and spermatogenesis, by analyzing the expression patterns of small RNAs in 6-, 18- and 30-month-old yak testis tissues.

**Results:**

A total of 737 known and 359 novel miRNAs were obtained from 6-, 18- and 30-month-old yak testes. In all, we obtained 12, 142 and 139 differentially expressed (DE) miRNAs in 30- *vs*. 18-, 18- *vs*. 6-, and 30- *vs*. 6-month-old testes, respectively. Gene Ontology (GO) annotation and Kyoto Encyclopedia of Genes and Genomes (KEGG) pathway analysis of all DE miRNA target genes revealed *BMP2*, *TGFB2*, *GDF6*, *SMAD6*, *TGFBR2* and other target genes as participants in different biological processes, including TGF-β, GnRH, Wnt, PI3K–Akt, MAPK signaling pathways and several other reproductive pathways. In addition, quantitative reverse transcriptase-polymerase chain reaction (qRT-PCR) was used to detect the expression of seven randomly selected miRNAs in 6-, 18- and 30-month-old testes, and the results were consistent with the sequencing data.

**Conclusions:**

The differential expression of miRNAs in yak testes at different development stages was characterized and investigated using deep sequencing technology. We believe that the results will contribute to further understanding the functions of miRNAs in regulating the development of yak testes and improving the reproductive performance of male yaks.

**Supplementary Information:**

The online version contains supplementary material available at 10.1186/s12917-023-03602-7.

## Background

Yaks are known to inhabit the area centered on the Qinghai–Tibet Plateau at an altitude of 2000 to 5000 m and are one of the most important livestock in these high-altitude areas [1]. More than 14 million yaks provide the basic resources, such as meat, milk, transportation and hides for tented accommodation, to the Tibetans and other nomadic pastoralists in high-altitude environments [2]. However, reproductive problems such as late sexual maturity, long calving intervals, and inconspicuous estrus expression have restricted the development of the yak industry and the living standards of local inhabitants [3]. The testis is an important reproduction organ in male animals, and its primary function is to produce sperms and androgens [4]. Because normal testicular development is highly crucial for species breeding, it is essential to study testicular development in early puberty yaks to improve semen quality and promote yak production.

MicroRNAs (miRNAs) are a class of endogenous small non-coding RNAs that are 1 to 24 nucleotides long. They are known as post-transcriptional repressors that regulate gene expression by inhibiting mRNA translation or regulating mRNA degradation post-transcriptionally [5]. Over the past few decades, expression profiling studies by miRNA microarray or small RNA sequencing have demonstrated the exclusive or preferential expression of several miRNAs in the testis or germ cells of humans, mice and mammals [6]. In mammals, miRNAs regulate a variety of physiological processes, including cell proliferation, tumorigenesis, hormone secretion, metabolism and reproductive regulation [7]. In addition, numerous functional studies have demonstrated the function of miRNAs in fertility and development. In particular, there is substantial evidence that miRNAs are involved in several aspects of reproductive physiology, including testicular development, spermatogenesis, and embryonic development [8–11]. A study on miRNAs in goats, the researchers identified 128 conserved miRNAs and speculated them to be involved in the development and meiosis in dairy goat testis [12]. Similarly, a recent study showed that LOC105611671 targeted oar-miR-26a to regulate the expression of FGF9, thereby promoting testicular steroidogenesis in Hu sheep Leydig cells [13]. The overexpression of bta-miR-136b inhibited the proliferation of bovine male germ line stem cells and promoted cell apoptosis [14]. A study in 2018, used Illumina HISeq and bioinformatics analysisand identified 50 differentially expressed (DE) known miRNAs and 11 DE new miRNAs in the testis of cattleyak and yak, speculating that these miRNAs could be related to the spermatogenic arrest in cattleyak [15]. Similarly, the differential expression of miRNAs and mRNAs in the testis of Tianzhu white yak 30 days after birth, and 2- and 4-years-old identified and analyzed 589 DE miRNAs by high-throughput sequencing. In addition, a functional analysis found miR-574 targeted AURKA and contributed to yak testicular development and reproduction [16]. However, identification of miRNAs involved in testis development and the underlying molecular mechanism have not been studied in yaks.

In the present study, we used deep sequencing technology to characterize and investigate the differential expression of miRNAs in yak testis at different developmental stages to understand the molecular regulatory mechanisms and identify key miRNA targets involved in yak testis development and spermatogenesis. This study will contribute to further understanding the functions of miRNAs in yak testis development and identify key miRNAs to improve male yak reproductive performance in the future.

## Results

### Overview of sequencing data

**Fig. 1:**
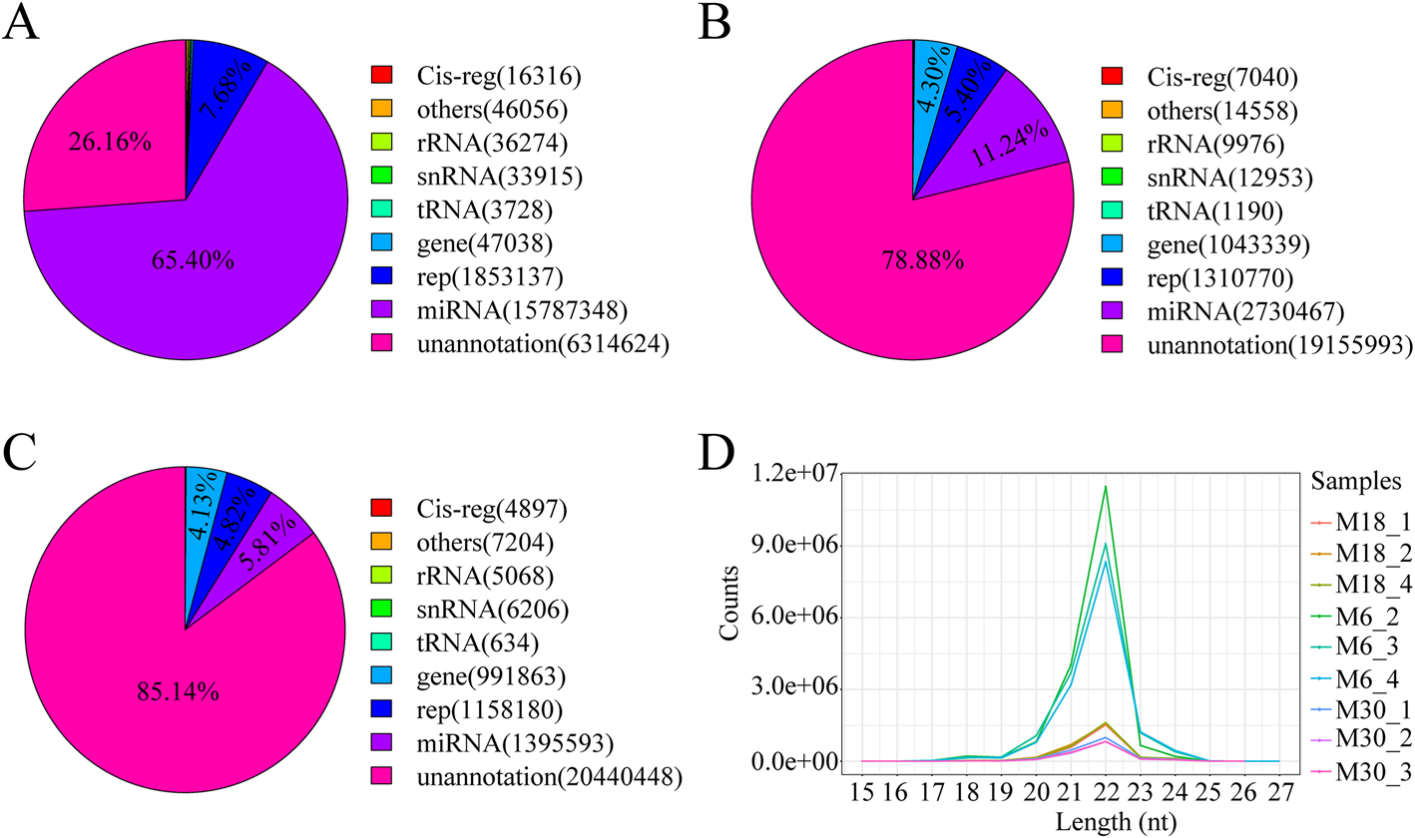
Identification and classification of miRNAs in yak testis. **A** Total number of unique sequences in the M6 library. **B** Total number of unique sequences in the M18 library. **C** Total number of unique sequences in the M30 library. **D** Length distribution and abundance of sequences in M6, M18 and M30 libraries

We analyzed small RNA populations in three libraries obtained from 6- (M6), 18- (M18) and 30- (M30) month-old yak testes using the Illumina Hiseq 2500 sequencing method. The miRNA sequence quality control results are shown in Supplementary Table S1. A total of 226.19 M raw reads were obtained, and following the removal of low-quality sequences, adapter sequences and discard of sequences shorter than 18 nt, 72.75 M, 72.65 M, and 72.49 M clean reads were obtained from the M6, M18, and M30 libraries, respectively, which were used for further analysis. A total of 3,402,676, 15,237,018 and 14,649,889 unique sRNA from M6, M18, and M30 testes were mapped to the yak reference genome, respectively (Table S2). All clean reads were aligned with the miRBase database and recorded as one of the known RNA categories according to their biogenesis and annotation (Fig. 1). As shown in Fig. 1A, 1B and 1C, the miRNAs in the M6 (65.4%) library constituted the largest proportion compared with M18 (11.24%) and M30 (5.81%) libraries. However, the highest proportions of unannotated small RNAs were present in M18 (78.88%) and M30 (85.14%) libraries, which represented other classes of small RNAs such as piRNAs. The read length distribution analyses of all small RNA libraries revealed that the dominant length of small RNA was 22 nt, accounting for at least 41.51% (Fig. 1D).

### Identification of known and novel miRNAs in yak testes

**Fig. 2:**
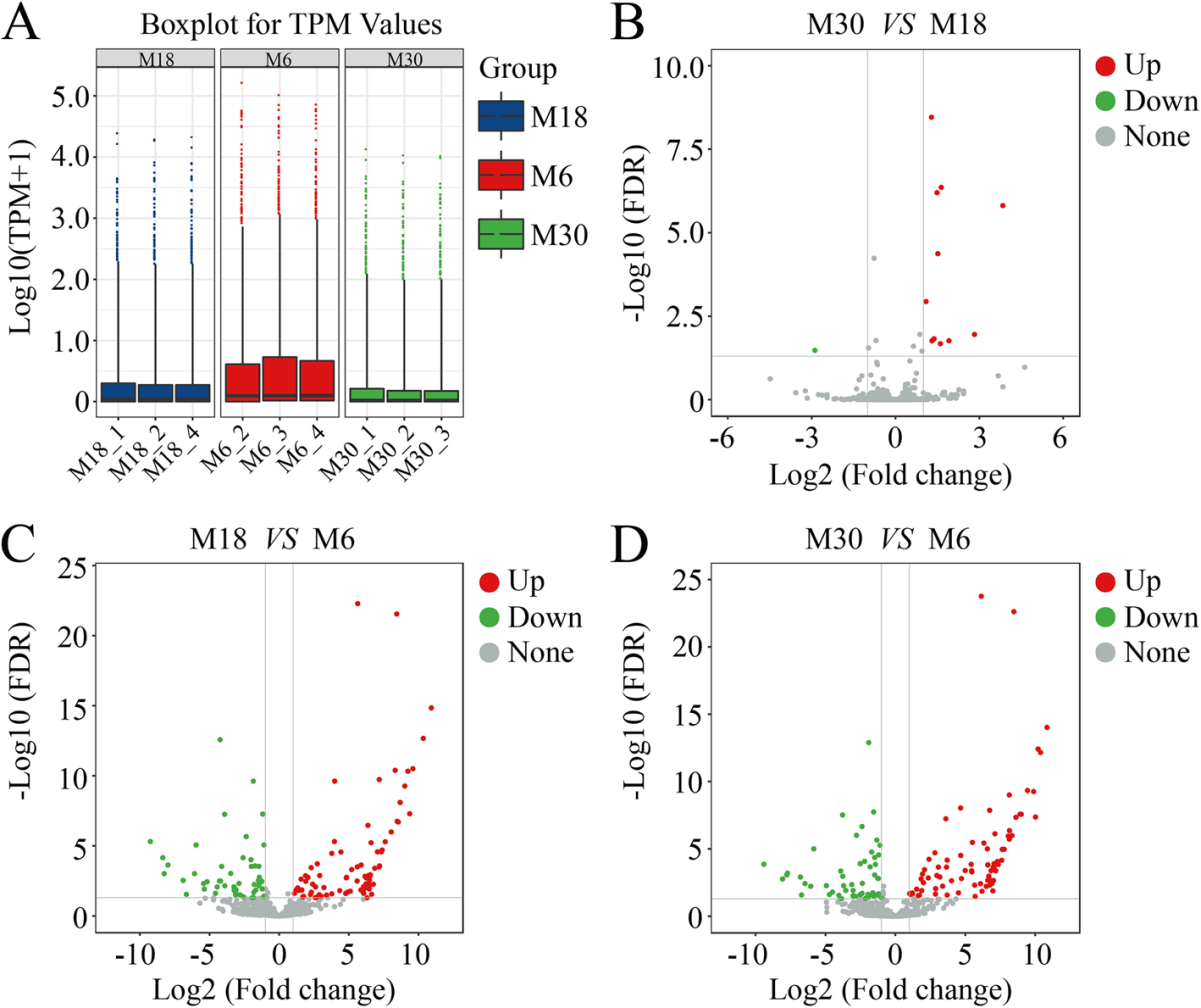
Comparative analysis of miRNAs in yak testis at different developmental stages. **A** The expression level of miRNAs in 6-, 18- and 30-month-old yak testes. **B** Differences of miRNA expression between M30 and M18 libraries. **C** Differences of miRNA expression between M18 and M6 libraries. **B** Differences of miRNA expression between M30 and M6 libraries. Red, green, and grayness dots in the graph represent transcripts that were significantly upregulated, downregulated and unchanged, respectively

To identify miRNAs in yak testis, the sequences obtained after removing small RNAs (including tRNA, snRNA, and rRNA) were compared with the miRBase database to obtain known miRNAs. Sequences that were not annotated into the miRBase database were predicted as novel miRNAs. A total of 737 known and 359 novel miRNAs were identified in the nine libraries (Table S3). Expression frequencies of each miRNA in the nine libraries varied widely, ranging from several to hundreds of thousands of sequence reads. The expression of these novel miRNAs was highly low, and their length ranged from 18 to 25 nt, with a distribution peak at 22 nt. Overall, the expression of most known miRNAs in 6-month-old yak testes was highest, followed by 18-month-old yak testes, and finally 30-month-old yak testes (Fig. 2A). The highest expressed members in all libraries were mir-2957, mir-143, let-7, mir-10 and mir-26 family members, each of which had more than 100,000 reads, except the mir-26 family in the M30 library.

### Differential expression of miRNAs in yak testis tissues

The transcriptional changes in miRNAs during the development of yak testis were analyzed using the DEG difference algorithm in the R package based on normalized reads. A comparison of miRNA expression profiles between all libraries is shown in Supplementary Tables S4-S6, using scatter plots (Fig. 2). In addition, volcano plots depict the pattern of DE miRNAs between all comparison groups, where only 12 differentially expressed miRNAs were identified between M30 and M18 testes; however, their expression was low (Fig. 2B). A total of 142 differentially expressed miRNAs were identified between M18 and M6, of which 90 were up-regulated and 52 were down-regulated (Fig. 2C). Similarly, 139 differentially expressed miRNAs were identified between M30 and M6, of which 85 were up-regulated and 54 were down-regulated (Fig. 2D).

### Target gene prediction for DE miRNAs

The target genes of miRNAs were predicted using the Miranda software. Next, the potential functional roles of these differentially expressed miRNAs were investigated. The potential targets of differentially expressed miRNAs were screened based on the criteria of total single-residue-pair match scores ≥ 150 and total energy ≤ –30 kcal/mol. In total, 474, 10,168 and 8385 target sites in 463, 7973 and 6677 target genes were predicted for 12, 142 and 139 miRNAs obtained from M30 vs. M18, M18 vs. M6, and M30 vs. M6, respectively. For most differentially expressed miRNAs, multiple distinct target genes existed; however, only one target gene was identified for certain differentially expressed miRNAs. In addition, certain target genes were targeted by multiple differentially expressed miRNAs. For instance, bta-miR-6526 putative targeted 39 genes and novel311_mature putative targeted 42 genes, whereas TGFB2 was only targeted by bta-miR-339b.

### Gene Ontology (GO) enrichment and KEGG pathway analysis of target genes

**Fig. 3:**
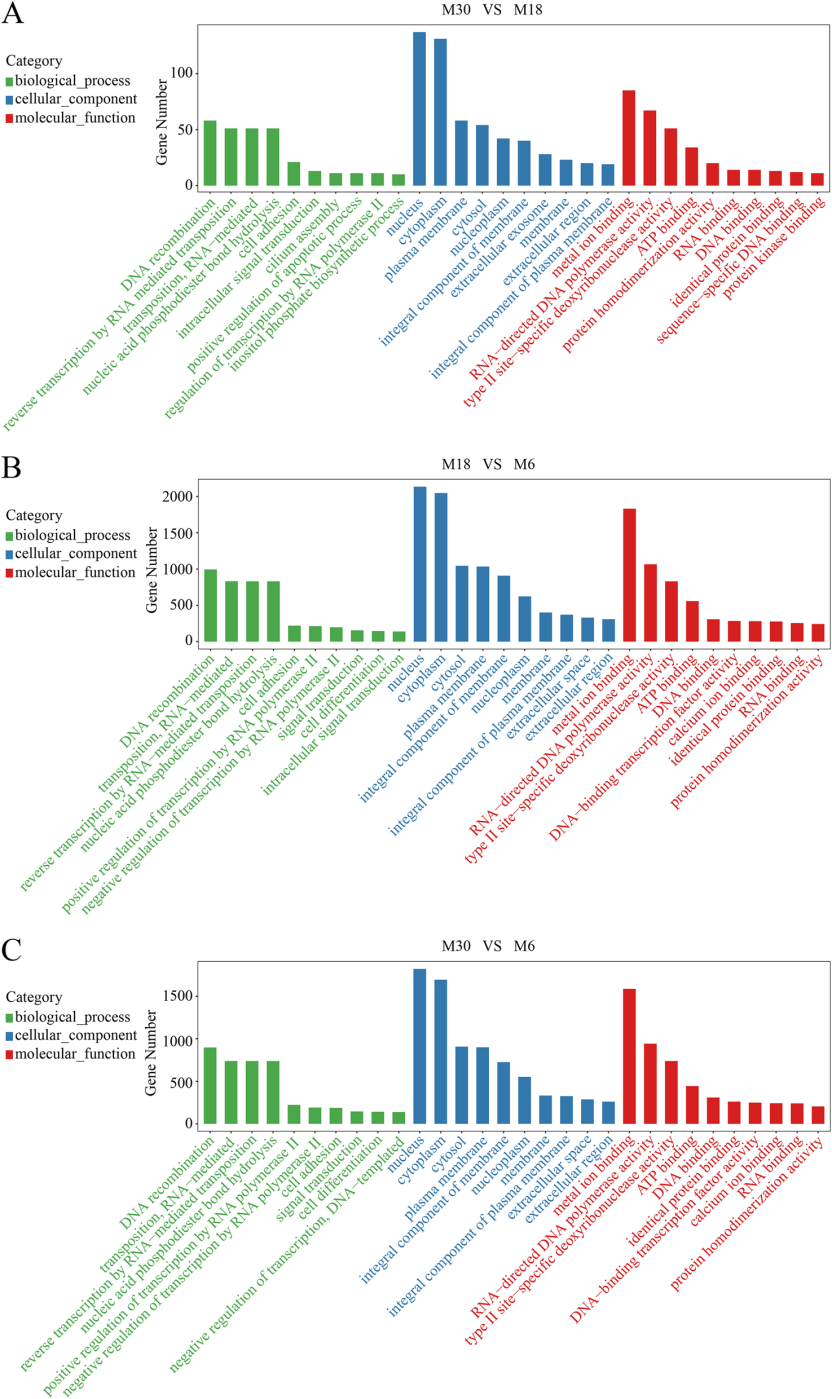
Gene ontology enrichment analysis of differentially expressed miRNA's target genes. **A** GO enrichment analysis of differentially expressed miRNA's target genes between M30 and M18. **B** GO enrichment analysis of differentially expressed miRNA's target genes between M18 and M6. **C** GO enrichment analysis of differentially expressed miRNA's target genes between M30 and M6

To better understand the functions of DE miRNAs in the yak testicular development, we next performed Gene Ontology (GO) and Kyoto Encyclopedia of Genes and Genomes (KEGG) enrichment analyses on candidate target genes of all DE miRNAs. The GO analysis results are shown in Fig. 3 and Tables S7-S9. Between M30 and M18, the target genes were significantly enriched in 224 BP terms, such as regulation of the androgen receptor signaling pathway, sperm capacitation, spermatid development, Sertoli cell differentiation and cholesterol metabolic processes. Figure 3A shows the top 10 GO terms in biological processes, cellular components and molecular functions, including inositol metabolic process, Sertoli cell differentiation, sperm annulus and sperm flagellum. Similarly, between M18 and M6, 410 significantly enriched GO terms for the target genes of DE miRNAs, in which cell adhesion, plasma membrane and ATP binding were the top terms involved in biological processes, molecular functions and cellular components, respectively (Fig. 3B). Between M30 and M6, 410 significantly enriched GO terms for the target genes of DE miRNAs, in which cell adhesion, plasma membrane and vascular endothelial growth factor-activated receptor activity were the top terms involved in biological processes, molecular functions and cellular components, respectively (Fig. 3C).

**Fig. 4:**
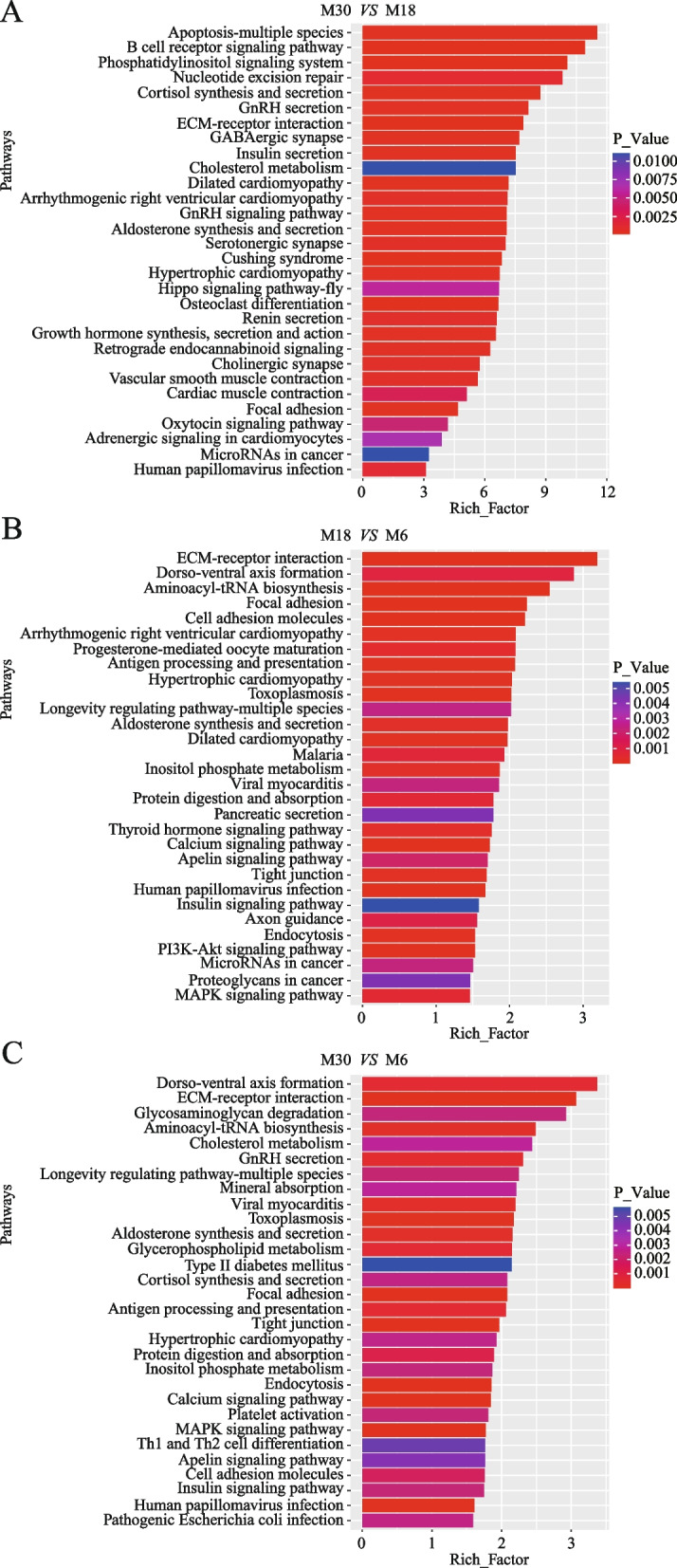
KEGG analysis of differentially expressed miRNA's target genes. **A** KEGG analysis of differentially expressed miRNA's target genes between M30 and M18. **B** KEGG analysis of differentially expressed miRNA's target genes between M18 and M6. **C** KEGG analysis of differentially expressed miRNA's target genes between M30 and M6. The vertical and horizontal axes represent the enrichment pathways and Rich factors of these path of each pathway are color coded

The KEGG pathway annotation revealed that the target genes of DE miRNAs in the M30 and M18 comparison groups were significantly enriched in 39 pathways, such as the GnRH signaling pathway, growth hormone synthesis secretion and action, cAMP signaling pathway, MAPK signaling pathway and PI3K–Akt signaling pathway (Fig. 4A and Table S10). The five enriched pathways related to testicular development we focused on included the GnRH signaling pathway, TGF-beta signaling pathway, MAPK signaling pathway, PI3K–Akt signaling pathway and Wnt signaling pathway. Between M18 and M6, the target genes were significantly enriched in 49 pathways, such as the PI3K–Akt signaling pathway, MAPK signaling pathway, thyroid hormone signaling pathway and GnRH secretion (Fig. 4B and Table S11). The six enriched pathways related to testicular development we focused on included the PI3K–Akt signaling pathway, MAPK signaling pathway, GnRH secretion, TGF–beta signaling pathway, Ras signaling pathway and AMPK signaling pathway. Similarly, between M30 and M6, the target genes were significantly enriched in 49 pathways, such as the MAPK signaling pathway, calcium signaling pathway, aldosterone synthesis and secretion, GnRH secretion, PI3K–Akt signaling pathway, Ras signaling pathway and ABC transporters (Fig. 4C and Table S12). These target genes were also enriched in pathways related to testicular development, including the PI3K–Akt signaling pathway, MAPK signaling pathway, GnRH secretion pathway, Wnt signaling pathway, Ras signaling pathway and AMPK signaling pathway.

### Quantitative RT-PCR validation

**Fig. 5:**
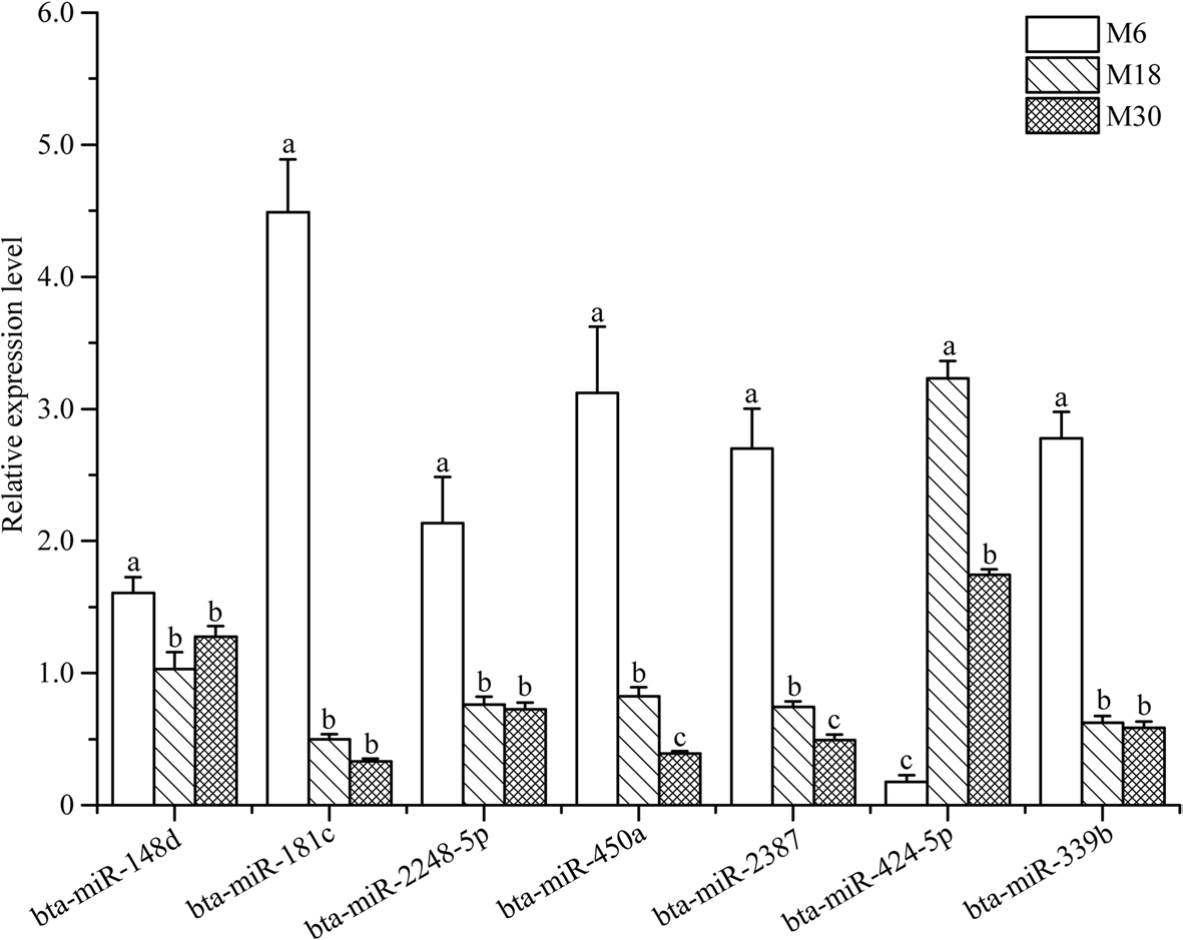
Validation of the expression of differentially expressed miRNAs by qRT-PCR. Different capital letters indicate that means differ significantly (*P* < 0.05)

Seven known yak miRNAs including bta-miR-148d, bta-miR-181c, bta-miR-2284 h-5p, bta-miR-450a, bta-miR-2387, bta-miR-424-5p and bta-miR-339b were randomly selected to validate the RNA-seq data. As shown in Fig. 5, qRT-PCR was performed in M30, M18 and M6 groups to detect the miRNA expression, respectively. The qRT-PCR results showed a similar expression trend to that obtained from small RNA-seq.

## Discussion

The performance of thereproductive system in male mammals is largely established during the early stages of testicular development, where differentiation of somatic cells to Sertoli cells in the early gonad initiates male-specific development and guides germ cells to the spermatogenic lineage [4]. Therefore, testicular development and spermatogenesis are key processes affecting the reproductive efficiency in male yaks. Moreover, several functional genes have been reported to regulate testicular development and spermatogenesis in yaks [17–19]. Recently, miRNAs have been implicated in gene regulation by repressing the translation or degradation of target mRNAs [20–22]. Since the discovery of miRNAs and their functions in regulating gene expression, several scholars have further studied miRNAs and found them to be involved in multiple processes of testicular development and spermatogenesis, such as testosterone hormone synthesis, cell proliferation, spermatocyte meiosis and spermiogenesis of haploid spermatids [23–25]. However, only a few studies have been conducted to characterize miRNAs involved in testicular development in early puberty yaks. In this study, we used the Illumina Solexa technology to sequence small RNAs in testicular tissues of 6-, 18- and 30-month-old male yak, analyze DE miRNAs, predict novel miRNAs, and perform GO enrichment and KEGG pathway analysis of target genes in three miRNA libraries.

Male yaks aged 6, 18, and 30 months, corresponding to infant, adolescent and sexual maturity stages, respectively, displayed a dynamic process of testicular development. Only 12,142 and 139 known DE known miRNAs were identified in M30 vs. M18, M18 vs. M6 and M30 vs. M6 yak testes, respectively, due to the limited information in the yak miRBase. Novel miRNAs were predicted from bovine, rat, and mouse genomic miRBase; thus, more DE novel miRNAs were identified compared to known DE yak miRNAs. The novel DE miRNAs could contribute to the identification of key miRNAs in yak testicular development and provide more references for the yak miRBase database. Previous studies have reported that miR-196a, miR-183, miR-96 and miR-182 regulate the reproductive performance in male mammals, including the promotion of Sertoli cell proliferation, inhibition of Sertoli cell apoptosis, and spermatogenesis [26, 27]. In this study, the DE miRNAs, namely, bta-miR-196a, bta-miR-182, bta-miR-183 and bta-miR-96 were expressed significantly higher in the M30 group than in the M18 group. Combined with previous study results, we can deduce that bta-miR-196a, bta-miR-182, bta-miR-183 and bta-miR-96 could be included in the development in yak testes. A study in mouse Leydig cells demonstrated that miR-150 reduced the production of sex steroid precursors and testosterone in Leydig cells by inhibiting the expression of the STAR gene, whereas the knockdown of miR-150 promoted steroidogenesis in Leydig cells [28]. The target genes of miR-155 are involved in the GnRH, MAPK and Wnt signaling pathways and affect the motility of sperm [29]. MiR-98 targets three apoptotic genes, namely, TP53, CASP3 and FASL, thereby disrupting the functions of Sertoli and promoting germ cell apoptosis [30]. Similarly, in the present study, the expression of miR-150, miR-155 and miR-98 was significantly decreased along with the testicular development of yak. Therefore, these miRNAs could be involved in yak testicular development. However, specific functions of these miRNAs in yak testes need further exploration and validation.

The GO annotation and KEGG pathway analysis can provide a detailed and comprehensive understanding of the functions of target genes of DE miRNAs from the aspects of cellular components, biological processes, molecular functions and the involved pathways. The GO and KEGG analyses indicated that the target genes of the DE miRNAs were involved in different biological processes, including growth and development, reproduction, cell proliferation, hormone secretion and several other metabolic processes. Interestingly, TGF-β, GnRH, PI3K-Akt, MAPK, Wnt and AMPK signaling pathways are widely considered to have regulatory effects on reproduction.

GnRH further regulates the development of testicles by promoting gonadotropin secretion and testosterone synthesis [31]. The most multifunctional miRNAs controlling the target genes in the GnRH signaling pathway include bta-miR-1307, bta-miR-1388-3p, bta-miR-150, bta-miR-326 and bta-miR-339b. Each of them regulated two to five genes in the canonical GnRH signaling pathway. PI3K–Akt signaling pathway can directly or indirectly maintain and promote spermatogenesis by regulating the proliferation, survival and anti-apoptosis of immature Sertoli cells, mature Sertoli cells, spermatogonia stem cells and spermatogenic cells [32]. A total of 47 genes targeted by 26 DE miRNAs were enriched in the PI3K–Akt signaling pathway. The target genes INSR and IGF1R of these DE miRNAs are important regulators of mitochondrial architecture and biogenesis markers in Leydig cells, with significant roles in mitochondrial biogenesis in steroidogenic cells [33]. In addition, the target gene PRLR of bta-miR-326 is a potential regulator of spermatogenesis, with significant regulatory effects to the proliferation of rat Sertoli cells [34]. Members of the TGF–β ligand superfamily are known to regulate the mammalian testicular development and spermatogenesis [35]. In this study, the DE miRNAs bta-miR-1307, bta-miR-326, bta-miR-339b, novo32_star and novel256_mature target multiple TGF-β family members were found to be enriched in the TGF–β signaling pathways, such as BMP2, TGFB2, SMAD6, GDF6, and TGFBR2, suggesting that these miRNAs are involved in yak testis development and spermatogenesis. In addition, 20 DE miRNAs including bta-miR-1307, bta-miR-1388-3p, bta-miR-326, bta-miR-339b, bta-miR-345-3p and novel32_sta targeted 25 genes gathered in the Wnt signaling pathway. In mice, the Wnt signaling pathway has been demonstrated to affect spermatogenesis by regulating the functions of Sertoli cells [36]. These miRNAs could be effective in the proliferation, differentiation and spermatogenesis of Sertoli cells. Evidence accumulated over the past decade has highlighted the major functions of the MAPK, AMPK and TGF–β signaling pathways during spermatogenesis, with MAPK signaling regulating germ cell proliferation, meiosis and Sertoli cell proliferation. Moreover, the AMPK and TGF–β signaling pathways affect the proliferation of Sertoli cells [37]. The most functional miRNAs controlling target genes in the TGF–β, GnRH, PI3K–Akt, MAPK, Wnt and AMPK pathways were bta-miR-1307, bta-miR-326, bta-miR-339b, novel32_star and bta-miR-7180. Each of them regulated multiple genes in different testicular development and spermatogenesis-related pathways. In the present study, the downregulation of bta-miR-135a, bta-miR-378 and bta-miR-184 accompanied the development of yak testis from juvenile to adulthood, indicating their significant role in promoting yak testicular development and spermatogenesis.

## Conclusions

In summary, we characterized the miRNA transcriptome at three developmental stages of the testis, from birth to sexual maturity, using Ashidan yaks. A total of 737 known miRNAs and 359 novel miRNAs were identified in nine libraries. The expression of these DE miRNAs in yak testis at different developmental stages was verified by RT-qPCR. The GO and KEGG pathway analyses on the target genes of all DE miRNAs in the library revealed the TGF-β signaling pathway, GnRH signaling pathway, Wnt signaling pathway and PI3K–Akt signaling pathway to be significantly enriched. We believe these results will contribute to the further understanding of the functions of miRNAs in yak testicular development and identify miRNAs to improve male yak reproductive performance in the future.

## Materials and methods

### Ethics statement

The study was approved by the Animal Administration and Ethics Committee of the Lanzhou Institute of Husbandry and Pharmaceutical Sciences of the Chinese Academy of Agricultural Sciences and Pharmaceutical Sciences and met the requirement of the institutional animal care and use committee (Permit no. 2019–002).

### Animals and sample preparation

Six-, 18- and 30-month-old male yak from the nucleus herds of Ashidan yak in the Datong Breeding Farm of Qinghai province were used. Three male yaks were selected for each age group; testes were obtained by veterinary surgery. Experimental samples were obtained after resecting the caudal epididymis, fat, and fascia around the testes. All samples were snap frozen in liquid nitrogen (− 196 °C), shipped to the laboratory and stored at − 80 °C for total RNA extraction.

### Small RNA library construction and sequencing

The total RNA in the testis tissue was extracted using the Trizol reagent (Invitrogen, USA). In total, 1 μg of the each sample was used to construct the small RNA library using TruSeq Small RNA Sample Prep Kits (Cat. no. RS-200–0012, Illumina, USA) following the manufacturer’s recommendations. Briefly, the total RNA was ligated to adapters at each end. Afterward, the adapter-ligated RNA was reverse transcribed to cDNA and PCR amplified. Library quality was assessed on the Agilent Bioanalyzer 2100 system using DNA High Sensitivity Chips. The libraries were finally sequenced using the Illumina HiSeq X Ten platform and 150 bp paired-end reads were generated. The small RNA sequencing was conducted by OE Biotech Co., Ltd. (Shanghai, China).

### Bioinformatic analysis

The Basic reads were converted into sequence data (also called raw data/reads) by base calling. Low quality reads were filtered and reads with 5’ primer contaminants and poly (A) were removed. Reads without 3’adapter and insert tags and those shorter than 15 nt or longer than 41 nt from the raw data were filtered to obtain the clean reads. For the primary analysis, the length distribution of clean sequences in the reference genome was determined. Non-coding RNAs were annotated as rRNAs, tRNAs, small nuclear RNAs (snRNAs), etc. These RNAs were aligned and subsequently subjected to the Bowtie [38] search against Rfam v.10.1 (http://www.sanger.ac.uk/software/Rfam) [39]. The known miRNAs were identified by aligning against the miRBase v22 database (http://www.mirbase.org/). The known miRNA expression patterns in different samples were analyzed [40]. Afterward, unannotated reads were analyzed by mirdeep2 to predict the novel miRNAs [41]. The corresponding miRNA star sequence and miRNA mature sequence were identified using the hairpin structure of a pre-miRNA and the miRBase database.

The expression of known and novel miRNAs was analyzed using TPM (transcripts per million; miRNAs normalized by TPM) [42]. The differential expression analysis of miRNAs between the two groups was performed using the DEG algorithm in the R package [43]. The P-values were adjusted using Benjamini and Hochberg’s method to control the false discovery rate (FDR). Differentially expressed miRNAs were defined when the adjusted FDR was < 0.05. In addition, we calculated the fold change (FC) between comparison groups using mean TPM values to define up-regulated (log_2_FC ≥ 1) and down-regulated (log_2_FC ≤ 1) miRNAs, respectively. The targets of DE miRNAs were predicted using the software Miranda, with the following parameters: single-residue-pair match scores ≥ 150, ΔG ≤  − 30 kcal/mol and demand strict 5' seed pairing [44]. GO enrichment and KEGG pathway enrichment analyses of DE miRNA-target-gene were performed using R considering the hypergeometric distribution [45–47].

### RNA-seq data validation

Seven known yak miRNAs were randomly selected and analyzed to validate the RNA-seq data using qRT-PCR. Real-time PCR was performed using LightCycler 480 II Real-time PCR Instrument (Roche, Swiss). Each sample was run in triplicate for analysis. The MicroRNA-specific primer sequences were designed in the laboratory and synthesized by TsingKe Biotech based on the microRNA sequences obtained from the miRBase database (Release 20.0). Primer information is shown in Table 1. The expression of microRNAs was normalized to U6 and was calculated using the 2^−ΔΔCt^ method [48].

**Table 1:** Details of primer sequences of miRNAs used for qRT-PCR

Name	Forward primer (5’ to 3’)	Reverse primer (5’ to 3’)
bta-miR-148d	GTTCTGTAGTGCACTGACTAA	Universal reverse *
bta-miR-181c	ATTCAACCTGTCGGTGAGTTT	Universal reverse *
bta-miR-2284 h-5p	CCAAAAGTTCGTTCGGGTTTA	Universal reverse *
bta-miR-450a	CTTTTGCGATGTGTTCCTAATAT	Universal reverse *
bta-miR-2387	AGGCCTGGCTTTGCAGCG	Universal reverse *
bta-miR-424-5p	GCAGCAGCAATTCATGTTTTGA	Universal reverse *
bta-miR-339b	CCTGTCCTCCAGGAGCTC	Universal reverse *
U6	CAAGGATGACACGCAAATTCG	Universal reverse *

^*^Universal reverse was provided by manufacture (miRcute Plus miRNA qPCR Detection Kit, TIANGEN BIOTECH, China)

## Supplementary Information

**Additional file 1:** Quality control of miRNA sequence.

**Additional file 2:** Read mapping summary of yak miRNA.

**Additional file 3:** The novel miRNAs and known miRNAs identified in all libraries.

**Additional file 4:** Differentially expressed miRNAs between M30 and M18.

**Additional file 5:** Differentially expressed miRNAs between M18 and M6.

**Additional file 6:** Differentially expressed miRNAs between M30 and M6.

**Additional file 7:** GO enrichment analysis of differentially expressed miRNA target genes between M30 and M18.

**Additional file 8:** GO enrichment analysis of differentially expressed miRNA target genes between M18 and M6.

**Additional file 9:** GO enrichment analysis of differentially expressed miRNA target genes between M30 and M6.

**Additional file 10:** KEGG enrichment analysis of differentially expressed miRNA target genes between M30 and M18.

**Additional file 11:** KEGG enrichment analysis of differentially expressed miRNA target genes between M18 and M6.

**Additional file 12:** KEGG enrichment analysis of differentially expressed miRNA target genes between M30 and M6.

## Data Availability

All data generated or analyzed during this study are included in this article.

## Abbreviations

miRNA: microRNA
DE: differentially expressed
GO: Gene ontology
KEEG: Kyoto encyclopedia of genes and genomes
qRT-PCR: Quantitative real-time PCR
M6: 6-month-old
M18: 18-month-old
M30: 30-month-old
sRNA: Small RNA
RIN: RNA integrity number
snRNAs: small nuclear RNAs
TPM: Transcript per million
FDR: false discovery rate
FC: fold change
